# Weather conditions and population level mortality in resource-poor settings – understanding the past before projecting the future

**DOI:** 10.3402/gha.v5i0.20010

**Published:** 2012-11-23

**Authors:** Joacim Rocklöv, Rainer Sauerborn, Osman Sankoh

**Affiliations:** 1 Department of Public Health and Clinical Medicine, Epidemiology and Global Health, Umeå University, Sweden; 2 Institute of Public Health University of Heidelberg, Germany; 3 INDEPTH Network, Accra, Ghana; 4 School of Public Health, University of the Witwatersrand, Johannesburg, South Africa; 5 Institute of Public Health University of Heidelberg, Germany

This supplement of *Global Health Action* shows how retrospective, longitudinal, population-based health data from several resource poor settings in Africa and Asia can enhance the understanding of the impact of the variability extremes of weather on mortality. This information is key to our understanding of the local impacts following from climate change, and to the development of adaptation strategies to mitigate such impacts. While the editors realize the strength of the empirical evidence generated, they also emphasize that this is merely a first step in the right direction ‘a proof of principle’ and that there are obvious needs to continue this work by widening and deepening the research efforts.

Severe and sometimes devastating consequences are considered to be associated with future climate change, with the largest potential impacts occurring in areas with the least means to adapt. Impacts on health range from those related to malnutrition, fresh water scarcity, and changes in the range and transmission of many infectious diseases to those directly related to weather or climate extremes, such as those caused by tropical storms, floods or heat waves (1).

Understanding future hazards and their health impacts is important for adaptation and mitigation policies. The former seeks to build climate change resilience into health systems; the latter brings the information on adverse health impacts into the climate policy debate as a further motivation to reduce net emissions (2).

The current understanding of health impacts from climate and weather is much better in high-income countries, while there is a strong belief that the impacts will be much more severe in low- and middle-income countries (LMICs). Although this belief is likely to be true, it still relies on limited empirical evidence. At present there is a lack of studies on the current health impacts from climate and weather on the population health in LMICs, particularly in rural Africa and Asia (1, 3, 4). This gap has immediate consequences for the understanding of the future local impacts on public health in these regions. One important factor related to the scarce evidence is the limited knowledge of even the simplest demographic and health statistics, such as the number of people living in a defined geographical area, the number of people that are born or deceased each year and the actual cause of death.

The INDEPTH network has collected high-quality standardized data on demographics and mortality for geographically defined populations over many years, particularly in regions of Africa and Asia (5). This effort has resulted in retrospective high-quality registers representative for regions where such information is rarely known (5). In addition to existing usage and implementation of health policies developed in collaboration with the INDEPTH network, this information can also provide a valuable basis for sound policy and decision-making with respect to climate change mitigation and adaptation. The papers in this supplement of *Global Health Action* describe the complex relationships between weather conditions and mortality in resource-poor settings of Africa and Asia. This is a first step in the process of learning from the past to understanding the future impacts of climate change.

In high-income areas, many studies have focused on the relationship between variability in weather, mortality and health, identifying stressors such as extreme heat and humidity (4, 6) and heavy downpours and floodings (7–10) as highly related to population health. Census and well-established retrospective health register data are commonly used. Analyses have been conducted to study, for example, the severe health impacts caused by extreme heat waves (11), susceptibility and vulnerability factors to temperature extremes (12), years of life lost due to heat and cold exposure (13), health impacts from drinking contaminated water associated with floodings and heavy rainfalls (7, 8), and the evaluation of adaptation measures such as warning systems and related action plans. Many of these studies have analysed the temporal relationship within time series of health conditions and weather exposures (14, 15). The rationale behind these studies is to estimate an expected event rate at a specific time of the year for a specific year, and then compare the observed event rate during specific weather conditions to the expected event rate forming a relative risk. In doing so, these studies incorporate trends and other time-varying factors known to be influential on the outcome necessary for predicting the expected event rates at specific time points, as well as variables related to weather conditions as explanatory variables. However, in LMICs, such relationships have been very sparsely studied using established and robust methodological considerations.

On these premises the INDEPTH Network, together with a number of south–south and north–south collaborating institutions, initiated a research and capacity-strengthening workshop in Nouna, Burkina Faso, in February 2011. Fifteen out of the 42 INDEPTH network member Health and Demographic Surveillance Systems (HDSSs) were represented by their analysts during a one-week workshop. The workshop was funded by UNESCO, Ghana, and UNESCO was represented at the workshop by Dr. Abdul Lamin. The facilitators of the workshop were from the INDEPTH Network secretariat, Accra, Ghana; Umeå University, Sweden; Heidelberg University, Germany; Columbia University, USA; and the Nouna Health Research Centre, Burkina Faso. The aim of the group was to work towards a special issue in the journal *Global Health Action* focusing on the relationship between weather conditions and mortality in HDSS areas. This activity goes under the name of CLIMO^1^ (Climate and Mortality).

Subsequently, a second follow-up workshop, funded by Doris Duke Charitable Foundation, was organised in Accra in May 2012. In between the two workshops, the analysts continued the work on their data with mentorship from the facilitators and commenced paper writing. The objective of the second workshop was to help the analysts develop their writing and scientific presentation skills in order to meet the standards of the upcoming scientific supplement in *Global Health Action*. This time, the facilitator group included the INDEPTH Network secretariat, Accra, Ghana; Umeå University, Sweden; Heidelberg University, Germany; London School of Hygiene and Medicine, UK; Virginia University, USA; and the Nouna Health Research Centre, Burkina Faso.

The facilitators laid out the objectives of the analyses and corresponding papers to be invited for submission to the supplement in *Global Health Action* in order to achieve a level of comparability of the individual papers. The analysts were asked to study the following: the relationship between weather and mortality in their HDSS registers; daily mortality (if possible) over a sufficiently long retrospective period; to incorporate the delayed effects associated with continuous weather exposures; investigate non-linearity; adjusting for confounding time-varying factors; to evaluate the model; and to stratify the analyses and presentations by age and sex. In addition, some researchers incorporated cause-specific mortality analyses, but this was not a uniform requirement.

The HDSS-associated analysts and researchers who contribute to the supplement have a wide geographical spread ranging from the west of sub-Saharan Africa to Bangladesh: Nouna HDSS (16), Burkina Faso; Navrongo HDSS (17), Ghana; Nairobi HDSS (18), Kenya; Rufiji HDSS (19), Tanzania; Vadu HDSS (20), India; AMK HDSS (21), Bangladesh; and Matlab HDSS (22), Bangladesh. All but one HDSS is situated in a rural area. The urban representation was based on data from the informal settlements of Nairobi. In general, income conditions in the regions are low or medium, with the African rural HDSSs representing the poorest regions.

The past, present and potentially future climatological contexts of the study areas are described in one of the papers (23). This paper makes an important contextual contribution to the supplement by describing the past to present changes in climate in the regions, as well as the projected changes in climate under different scenarios of greenhouse gas emissions. Hondula et al. (23) not only describe the changes in terms of temperature and rainfall trends in the study regions, but also transform and link the past to future climate in terms of the Köppen climate classification scheme. The results show potential substantial shifts in the climate type in a few of the study areas in the future, much larger than the observed past to present changes.

In their study, Egondi and co-workers describe the direct and delayed associations between cold and warm temperatures and the amount of rainfall on daily mortality in the informal settlements of Nairobi, Kenya, over the period 2003–2008 (18). Few studies, if any before, have studied an urban population under such poor living and sanitary conditions. The study reveals significant associations between both hot and cold temperatures and increasing amounts of rainfall, and for example deaths in children in infectious and non-communicable disease.

Azongo and co-workers have studied the relationship between daily mortality and temperature and rainfall including delays up to a few weeks between exposure and events (17) based on registers from the rural parts of the Kassena-Nankana District of northern Ghana, 1995–2010. In general, they have found the study population to be negatively affected by heavy rains, and that children and the elderly population appeared to suffer the most. The strongest associations were found with less than a week's lag.

In the study by Diboulo's group, the authors describe how the population mortality from northern Burkina Faso (bordering to the Saharan desert) is affected by different weather conditions on a daily scale over the period 1999–2009 (16). It appears children are most negatively affected by hot temperature, while the adult and elderly population appeared more susceptible to the effects related to heavy rainfalls. The authors discuss and present a U-shaped relationship between mortality and temperature in the elderly population, just like the ones found in most studies from more developed countries in the elderly population.

In a study from Rufiji, Tanzania, Mrema and co-workers the authors describe how rainfall and temperature on a monthly timescale are related to mortality (19). They describe how much of the annual seasonality in mortality that disappears after adjustment for the direct and lagged indicated effects from the meteorological conditions. They, like many of the other studies, reach the conclusion that children and the elderly population appear most sensitive to the effects associated with different weather conditions.

Ingole's group studied daily population level mortality in relation to weather in the rural area of Vadu, India, 2003–2010 (20). The weather conditions can get extremely hot during certain periods of the year in this region. The authors found that children appear most susceptible to hot temperatures, and most population subgroups to be negatively affected during heavy rainfalls. The indicated effect from rainfall was particularly strong among women.

The longest daily time series studied as part of the supplement is based on data from the Matlab and the Abhoynagar areas in Bangladesh, 1983–2009. Using the Matlab registers, Lindeboom and co-workers describe the associations between mortality, temperature, rainfall and cyclones (22). In this study population it appears that the relationship between mortality and temperature is non-linear and U-shaped, while the relationship with rainfall is rather weak. In addition, cyclones were found to be associated with increased mortality among adult women. The study based on the Abhoynagar registers shows weekly associations between mortality and weather (21). In this population it appears that low temperatures are more strongly associated with increases in mortality. However, heavy rainfall also positively correlated with increased mortality rates.

Overall, the results from the site-specific analyses are in line with many studies from more developed countries. They show that meteorological conditions are related to mortality days and/or weeks later. On the other hand, many of the studies also show that children under 5 years of age appear sensitive to mortality during extreme weather conditions. Some of the studies indicate a surprising absence of mortality impacts related to hot temperature. This may be related to the low prevalence of non-communicable diseases in the early stage of the epidemiological transitions (24). The studies also indicate that populations in sub-tropical areas of the world may suffer from cold-related mortality, which may be due to poor housing conditions and a lack of available fuel for heating. The populations studied appear vulnerable to heavy rainfall events with corresponding upsurges in mortality a few days or weeks after such events. Overall, the different studies point to local differences in impacts and susceptibilities, which is valuable for the development of local adaption strategies to current weather situations. It seems possible that simple measures such as improved hygiene and housing conditions may prove effective in reducing the weather-related burden of deaths in the study regions.

Relating the supplemental findings to the current body of evidence (mainly from developed regions), it is important to consider the state of demographic and epidemiological transitions in the study population. Most certainly, this is particularly important when considering future impacts of weather and climate change on non-communicable diseases, as such diseases are likely to become much more prevalent in the future (24).

Further studies are needed to study these relationships in more detail and to compare the results between the HDSS areas. For example, using cause-specific data and identifying factors related to increased vulnerability and susceptibility to get a better understanding of the causal pathways of these effects, and also on how to enhance the local resilience to the current environmental stressors, are important next steps. A mixed methods approach using qualitative studies of people's perceptions, current knowledge of weather and climate change-related health risks, and describing peoples means to adapt, together with quantitative studies describing and enumerating the health impacts would benefit both local and national policies and decision making by jointly describing a width and depth, and possibilities for adaptation in the communities. Studies should also be designed to better understand the global and local health impacts following from climate change. For example, using projections of disease and deaths based on epidemiological transitions and climate change scenarios can help the development of longer-term adaptation programmes and global policies.

The INDEPTH network provides a possibility to expand the observation period onwards. As where longer time series are and become readily available, it is important to study the health effects related to climate variability and change directly, for example in a detection and attribution to past to present climate change framework.

Further research should develop the use of climate and weather information in increasing public health preparedness (e.g. early warning systems) to, for example, extreme climate and weather events and infectious outbreaks. The INDEPTH network offers an opportunity for development and evaluation of such interventions based on the retrospective registers and prospective data collection.

The joint work of the INDEPTH network members, analysts and facilitators described in this supplement is therefore a first step in building capacity for enhancing the long-term resilience for the population in resource-poor settings of Africa and Asia to climate and climate change. Our collaborative effort using INDEPTH data to study the effects of weather on mortality is an important first step in the challenging and hitherto under-explored road of what the health impacts of climate change in low-income countries are and how populations in theses resource-poor countries can be protected from them.

*Joacim Rocklöv* Department of Public Health and Clinical Medicine Epidemiology and Global Health Umeå University, Sweden*Rainer Sauerborn* Department of Public Health and Clinical Medicine Epidemiology and Global Health Umeå University, Sweden Institute of Public Health University of Heidelberg, Germany*Osman Sankoh* INDEPTH Network, Accra, Ghana School of Public Health, University of the Witwatersrand, Johannesburg, South Africa Institute of Public Health University of Heidelberg, Germany

## Footnotes

1 A second branch of data analysis, currently underway, refers to the link between weather and migration patterns: hence the acronym for the full study CLIMIMO (Climate Migration and Mortality).
